# The Mediating Effect of Central Sensitization on the Relation between Pain Intensity and Psychological Factors: A Cross-Sectional Study with Mediation Analysis

**DOI:** 10.1155/2019/3916135

**Published:** 2019-04-08

**Authors:** Hayato Shigetoh, Yoichi Tanaka, Masayuki Koga, Michihiro Osumi, Shu Morioka

**Affiliations:** ^1^Department of Neurorehabilitation, Graduate School of Health Sciences, Kio University, Nara, Japan; ^2^Miura Internal Medicine Michiko Pediatrics Clinic, Marugame, Kagawa, Japan; ^3^Neurorehabilitation Research Center, Kio University, Nara, Japan

## Abstract

**Background:**

Central sensitization (CS) and psychological factors are associated with pain intensity; however, the mediating role of CS on the relation between psychological factors and pain intensity remains unclear.

**Objectives:**

We performed mediation analysis to investigate how CS mediates relation between psychological factors and pain intensity.

**Methods:**

Twenty patients with musculoskeletal pain were included in this cross-sectional study. Central sensitization inventory (CSI), one pain intensity-related outcome measure (Short-Form McGill Pain Questionnaire 2 (SFMPQ2)), and three psychological outcome measures (Hospital Anxiety and Depression Scale (HADS), Pain Catastrophizing Scale-4 (PCS), and Tampa Scale for Kinesiophobia-11 (TSK)) of all participants were assessed. The mediation analysis with a bootstrap sampling procedure was used to assess the indirect effects. The level of significance was set at 5%.

**Results:**

Mediation analysis showed that the HADS-anxiety, HADS-depression, and PCS had significant indirect effects on the pain ratings of CSI. Additionally, the direct effect was significant only for PCS.

**Conclusions:**

The relationship among anxiety symptoms, depression symptoms, and pain intensity was completely mediated by CS. Furthermore, the relationship between catastrophic thinking and pain intensity was partially mediated by CS. Our findings suggest that CS mediates relation between psychological factors and pain intensity, and CS-focused intervention may be important.

## 1. Introduction

Many musculoskeletal pain conditions, such as osteoarthritis [1, 2], low back pain [2, 3], and persistent neck pain [4, 5], are associated with hypersensitivity, which is induced by central sensitization (CS). The International Association for the Study of Pain defines CS as the “increased responsiveness of nociceptive neurons in the central nervous system to their normal or sub-threshold afferent input” [6]. This definition is used as the physiological concept of CS. Recently, the International Association for the Study of Pain released a new term, nociplastic, designed to be a third descriptor to be used instead of “central” or “central sensitization” [7]. Nociplastic pain is defined as “pain that arises from altered nociception despite no clear evidence of actual or threatened tissue damage causing the activation of peripheral nociceptors or evidence for disease or lesion of the somatosensory system causing the pain.” Nociplastic pain relates to hypersensitivity, including hyperalgesia. Nociplastic pain is used as the clinical concept of CS.

The central sensitization inventory (CSI) was recently developed as a comprehensive screening instrument for CS [8]. The use of CSI has also been recommended as one component of an algorithm to detect CS in patients with chronic pain [9], particularly in patients with musculoskeletal pain [10]. Several studies revealed that pain intensity was associated not only with psychological factors [10–12] but also with CSI score [3, 10, 11, 13, 14]. The CSI cutoff score has been recommended as a CSI score of >40, and it is based on the presence or absence of central sensitivity syndromes (CSSs) [15]. However, this is only a cutoff score, and even those with a CSI score of less than 40 may also have effects of CS. In fact, the previous study reported that the average score of CSI score was low in the Japanese version of CSI, and patients diagnosed with 1 or more CSSs scored lower on the CSI than 40 [13]. The CSI score in the previous study may be affected by the disease and the region of the subject. Patients who were referred to a multidisciplinary pain center, which specializes in the assessment and treatment of complex pain and psychophysiological disorders, including CSSs reported high CSI scores (>40) [15]. However, patients who were recruited from the community-based physiotherapy program reported low CSI scores (mean = 24.6; SD = 12.0) [16]. Focusing on the cultural differences, the Japanese mean score of the CSI (mean = 21.91; SD = 13.31) [13] was lower than the American (mean = 52.4; SD = 14.3) [15] and Spanish (mean = 24.6; SD = 12.0) [16] samples. Thus, although there is a cutoff score of CSI, it may be better to pay attention to the amount of numerical values not cutoff score, as the CSI score may also be affected by the cultural differences and disease.

Studies, such as those cited above, did not determine how CS and psychological factors influence pain intensity in any relationship [3, 10–14]. Psychological factors are reportedly associated with pain intensity, but pain intensity is not always increased by negative emotions [17, 18]. For instance, anxiety reportedly has direct correlation with pain intensity [10–12], but not always has correlation with pain [17]. Also in depression, catastrophic thinking, and kinesiophobia, several reports suggested that pain intensity was related to these psychological factors [10–12], but several reports also suggest that pain intensity was not always correlated with these psychological factors [18]. We thought that the existence of CS will affect these inconsistent reports, and we hypothesized that CS mediates relationships between psychological factors and pain intensity. However, the mediating role of CS on the relationship between psychological factors and pain intensity has never been investigated. Mediation analysis could help determine how CS and psychological factors modify pain intensity in any relationship, and we believe that this knowledge will contribute to the selection of optimal treatments based on the pathology of pain-related CS in clinical settings.

The primary aim of this study was to reveal how CS mediates relation between psychological factors and pain intensity. We hypothesized that CS mediates relation between psychological factors and pain intensity.

## 2. Methods

### 2.1. Participants

In total, 20 patients were recruited from an orthopedic clinic. Patients aged between 16 and 86 years and having musculoskeletal pain, such as pain involving the neck, shoulder, low back, or knee, were included (Table 1). Previous studies have reported that CS occurs in multiple sites such as the knee [1, 2], lumbar region [2, 3], and neck [4, 5], so we did not limit the pain site. Therefore, we would like to investigate the effect of CS on musculoskeletal pain without identifying sites and diseases susceptible to CS. Also, we did not limit the pain duration to investigate various pain conditions. Exclusion criteria were patients diagnosed with brain or spinal cord injury, neurological disease, or dementia. The study protocol conformed to the Declaration of Helsinki. The participants provided written informed consent before the study began. This study was approved by the ethics committee of Kio University Health Sciences Graduate School (approval no. H30-06).

### 2.2. Procedure

Demographic data (age, sex, pain area, and duration), CSI, one measure of pain intensity-related outcomes (Short-Form McGill Pain Questionnaire 2 (SFMPQ2) [19], and three measures of psychological outcomes (Hospital Anxiety and Depression Scale (HADS) [20], Pain Catastrophizing Scale-4 (PCS) [21], and Tampa Scale for Kinesiophobia-11 (TSK) [22]) of all participants were assessed.

The Japanese version of CSI was used to assess CS [13]. CSI consists of 2 parts. Part A is a questionnaire comprising 25 self-report items and is used to assess health-related symptoms that are common to CSSs. Part B was not used in this study. Higher scores indicate more severe CS. CSI had good internal consistency (Cronbach's *α* = 0.89). A factor analysis yielded 5 major factors [13].

SFMPQ2 was used to assess pain intensity [19] and includes items that assess 22 qualities of pain and the intensity of each quality on an 11-point numerical rating scale. The total score is calculated from the sum of the 22 items. Higher scores indicate more severe pain. SFMPQ2 had good internal consistency (SFMPQ2-total: Cronbach's *α* = 0.86) [19]. There were significant correlations between SFMPQ2-total and other functional assessments (VAS: *ρ* = 0.54, SFMPQ-total: *ρ* = 0.79) [19].

HADS was used to assess anxiety and depression as one of psychological factors [20]. HADS contains 14 items and 2 subscales. The two subscales independently assess depression and anxiety. Higher scores indicate more severe anxiety and depression. HADS-anxiety had good internal consistency (HADS-anxiety: Cronbach's *α* = 0.80), and HADS-depression had not good internal consistency (HADS-depression: Cronbach's *α* = 0.50–0.61) [20]. The correlations of the HADS-anxiety scores and the state-trait anxiety inventory (STAI) were 0.63–0.65. The correlations of the HADS-depression scores and Zung's self-rating depression scale (SDS) were 0.46–0.50 [20].

PCS-4 was used to assess catastrophic thinking as one of psychological factors [21]. PCS-4 is a shorter version of a 13-item PCS and contains 4 items. Higher scores indicate more severe catastrophic thinking. PCS-4 had good internal consistency (Cronbach's *α* = 0.86) [21]. There were significant correlations between PCS-4 and PCS-13 (*r* = 0.96) [21].

TSK-11 was used to assess kinesiophobia as one of psychological factors [22]. TSK-11 is a shorter version of a 17-item TSK and contains 11 items. Higher scores indicate more severe kinesiophobia. TSK-11 had good internal consistency (Cronbach's *α* = 0.74–0.87) [22]. A factor analysis yielded 2 major factors [22].

### 2.3. Statistical Analysis

Mediation analysis was performed to assess the indirect effects of CSI on the relationship between psychological factors and pain intensity. The CSI was used as a continuous variable not as a dichotomous (presence or absence of CS by a cutoff score), because it was difficult to distinguish CS clearly into dichotomous, and the CSI was used as a continuous variable in this study for mediation analysis. To assess mediation, the following conditions had to be met [23]. (a) The effect of the independent variable on the dependent variable without the mediated variable is evaluated. (b) The effect of the independent variable on the mediated variable is assessed. (c) The role of both the independent and mediated variables on the dependent variable is evaluated. A bootstrap sampling procedure, as recommended for small sample sizes, was used to determine the significance of indirect effects [24]. This process involved using the sample as a population reservoir from which a large number of random samples were drawn and continuously replaced so that they had an equal likelihood of being randomly selected on all subsequent drawings. In the present study, we specified 1000 bootstrap iterations, as previously described [24]. In the mediation model used, the bootstrapped values of the 95% confidence interval that do not contain 0 between their lower and upper limits were considered to be significant mediators [25]. The statistical analyses were performed with HAD [26]. The level of significance was set at 5%.

## 3. Results

### 3.1. Sample Characteristics

A summary of the demographic characteristics and clinical profile of all participants is provided in Table 1. In total, the mean score of CSI-J was 24.0 ± 12.7 (mean ± SD).

### 3.2. Mediation Analysis

**Figure 1:**
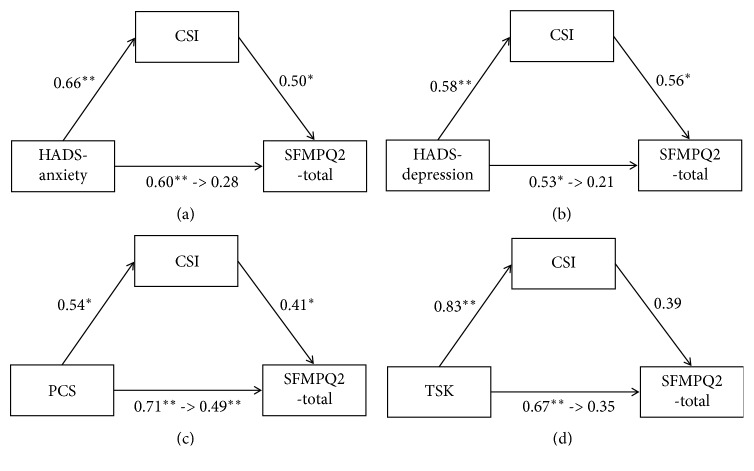
Central sensitization inventory (CSI) mediates the relationship between psychological factors and Short-Form McGill Pain Questionnaire 2 (SFMPQ2). Standardized betas are shown. (a) Hospital Anxiety and Depression Scale (HADS)-anxiety is the independent variable. (b) HADS-depression is the independent variable. (c) Pain Catastrophizing Scale-4 (PCS-4) is the independent variable. (d) Tampa Scale for Kinesiophobia-11 (TSK-11) is the independent variable. ^*∗*^*p* < 0.05; ^*∗∗*^*p* < 0.01.

We investigated whether CSI mediated the relationship between psychological factors and pain intensity. The tested model is illustrated in Figure 1.  Table 2 shows that the direct effects of the hypothesized model were statistically significant only for PCS. In addition, it shows that the 95% BC bootstrapped CI for the indirect effects of HADS-anxiety (95% BC bootstrapped CI, 0.208–7.176 with 1000 resamples), HADS-depression (95% BC bootstrapped CI, 0.714–6.780 with 1000 resamples), and PCS (95% BC bootstrapped CI, 0.437–9.589 with 1000 resamples) on pain ratings of CSI was significantly different from zero. However, the 95% BC bootstrapped CI for the indirect effect of TSK (95% BC bootstrapped CI, −0.367 to 5.155 with 1000 resamples) was not significantly different from zero. These results revealed that the relationships between PCS and SFMPQ2-total were partially mediated by CSI. In addition, the relationship among HADS-anxiety, HADS-depression, and SFMPQ2-total was completely mediated by CSI.

## 4. Discussion

We used mediation analysis to investigate the relationship between pain intensity of CS and psychological factors. The results showed that the relationship among anxiety symptoms, depression symptoms, and pain intensity was completely mediated by CS. Moreover, the relationship between catastrophic thinking and pain intensity was partially mediated by CS.

Several cross-sectional studies showed that both psychological factors and CS affected pain intensity [3, 10–14]. Similarly, the present study showed that all psychological factors had significant total effects on pain intensity. Based on the results of the mediation analysis, although only catastrophic thinking had a direct effect on pain intensity, psychological factors were mediated in pain through CS. Thus, psychological factors apparently affected pain intensity, but CS directly affected the pain intensity in practice.

This is the first study to demonstrate that the effects of psychological factors (i.e., anxiety, depression, and catastrophic thinking) on pain intensity were mediated by CS. This may be biologically plausible because high CS scores indicate the dysfunction of supraspinal processing [27]. Previous studies have reported that negative emotion impairs the descending inhibitory pathways [28, 29]. For example, one previous study reported that the diffuse noxious inhibitory control was impaired in patients with chronic pain who have depression [29]. Thus, pain modulation may be induced not only by negative emotion but also by central nervous system distortion. In clinical settings, consideration should be given to CS, which is modified by psychological factors and may be effective for pain treatment. A previous study reported that rehabilitation exercises were effective on CS, pain, disability, and fear avoidance belief in patients with chronic nonspecific low back pain [3], suggesting that rehabilitation exercises improve CS. Progress in the study regarding rehabilitation is expected in the future.

This study showed that catastrophic thinking directly affected pain without mediation through CS. This may be biologically plausible because catastrophic thinking has cognitive factors such as attention to pain. A previous study reported that even with lower anxiety and depression scores, higher catastrophic thinking affected the pain intensity [30], thereby possibly indicating that catastrophic thinking affects pain intensity as a cognitive factor and not as an affective factor. Moreover, affect and attention changed pain intensity by different descending inhibitory pathways [28]. The attention to pain activated the pain pathway that is associated with pain intensity. Thus, attention to pain, such as the careful catastrophic thinking, may increase pain intensity by activating the pain pathway. Although we did not directly evaluate these biochemical data, such mechanisms can be assumed to be involved. In clinical settings, considering the attention to pain may be effective for the pain is modified by the catastrophic thinking.

This study had several limitations. First, the outcomes of CS were merely those measured by CSI. Second, the sample size was relatively small. However, we adopted a bootstrap sampling procedure to determine the significance of the indirect effects. Third, we could not determine the mechanisms underlying the relationship between pain intensity of CS and psychological factors because this study did not measure neurotransmitter levels.

## 5. Conclusion

To our knowledge, this is the first study to investigate mediation by CS for the effects of psychological factors on pain intensity. The relationship among anxiety symptoms, depression symptoms, and pain intensity was completely mediated by CS. Additionally, the relationship between catastrophic thinking and pain intensity was partially mediated by CS. Our results suggest that CS mediates relation between psychological factors and pain intensity and CS-focused intervention may be important.

## Figures and Tables

**Table 1:** Characteristics of the participants.

	Mean (SD) or *N* (%)
Age (years)	67.5 (15.6)
Gender (female)	12 (60)
Pain area	
Neck	3 (15)
Low back	11 (55)
Shoulder	4 (20)
Knee	2 (10)
Pain duration (months)	24.3 (41.4)
Central Sensitization Inventory (CSI)	24.0 (12.7)
Short-Form McGill Pain Questionnaire 2 (SFMPQ2)-total	41.6 (35.5)
Hospital Anxiety and Depression Scale (HADS)-anxiety	5.9 (4.3)
Hospital Anxiety and Depression Scale (HADS)-depression	6.3 (3.7)
Pain Catastrophizing Scale (PCS)-4	6.9 (2.0)
Tampa Scale for Kinesiophobia (TSK)-11	13.1 (6.2)

**Table 2:** Mediation analysis: the role of CSI as a mediator.

Path/effect	*β*	SE	*p* value/95% BCCI
*a* HADS-anxiety ⟶ CSI	0.659	0.522	0.002
*b* CSI ⟶ SFMPQ2-total	0.496	0.632	0.043
*c* (direct effect) HADS-anxiety ⟶ SFMPQ2-total	0.277	1.861	0.239
*c*′ (total effect) HADS-anxiety ⟶ SFMPQ2-total	0.604	1.540	0.005
*a* × *b* (indirect effect) HADS-anxiety ⟶ SFMPQ2-total	0.327	1.729	(LL = 0.208, UL = 7.176)

*a* HADS-depression ⟶ CSI	0.578	0.655	0.008
*b* CSI ⟶ SFMPQ2-total	0.559	0.592	0.018
*c* (direct effect) HADS-depression ⟶ SFMPQ2-total	0.206	2.015	0.345
*c*′ (total effect) HADS-depression ⟶ SFMPQ2-total	0.530	1.896	0.016
*a* × *b* (indirect effect) HADS-depression ⟶ SFMPQ2-total	0.323	1.501	(LL = 0.714, UL = 6.780)

*a* PCS ⟶ CSI	0.537	1.252	0.015
*b* CSI ⟶ SFMPQ2-total	0.414	0.485	0.029
*c* (direct effect) PCS ⟶ SFMPQ2-total	0.492	3.053	0.012
*c*′ (total effect) PCS ⟶ SFMPQ2-total	0.715	2.889	0.0004
*a* × *b* (indirect effect) PCS ⟶ SFMPQ2-total	0.222	2.362	(LL = 0.437, UL = 9.589)

*a* TSK ⟶ CSI	0.830	0.269	0.00001
*b* CSI ⟶ SFMPQ2-total	0.390	0.859	0.223
*c* (direct effect) TSK ⟶ SFMPQ2-total	0.347	1.759	0.277
*c*′ (total effect) TSK ⟶ SFMPQ2-total	0.671	0.996	0.001
*a* × *b* (indirect effect) TSK ⟶ SFMPQ2-total	0.324	1.395	(LL = −0.367, UL = 5.155)

SE, standard error; BC, bias corrected; CI, confidence interval; LL, lower limit; UL, upper limit.

## Data Availability

The data used to support the findings of this study are available from the corresponding author upon request.

## References

[1] Lluch E., Nijs J., Courtney C. A. (2018). Clinical descriptors for the recognition of central sensitization pain in patients with knee osteoarthritis. *Disability and Rehabilitation*.

[2] Staud R. (2011). Evidence for shared pain mechanisms in osteoarthritis, low back pain, and fibromyalgia. *Current Rheumatology Reports*.

[3] Bid D. D., Soni C. N., Yadav S. A., Rathod V. P. (2017). A study on central sensitization in chronic non-specific low back pain. *Indian Journal of Physiotherapy and Occupational Therapy-An International Journal*.

[4] Sterling M., Treleaven J., Edwards S., Jull G. (2002). Pressure pain thresholds in chronic whiplash associated disorder: further evidence of altered central pain processing. *Journal of Musculoskeletal Pain*.

[5] Sterling M., Jull G., Vicenzino B., Kenardy J. (2003). Sensory hypersensitivity occurs soon after whiplash injury and is associated with poor recovery. *Pain*.

[6] Loeser J. D., Treede R.-D. (2008). The Kyoto protocol of IASP basic pain Terminology^☆^. *Pain*.

[7] Andrews N. (2018). *What’s in a Name for Chronic Pain?*.

[8] Mayer T. G., Neblett R., Cohen H. (2012). The development and psychometric validation of the central sensitization inventory. *Pain Practice*.

[9] Nijs J., Apeldoorn A., Hallegraeff H. (2015). Low back pain: guidelines for the clinical classification of predominant neuropathic, nociceptive, or central sensitization pain. *Pain Physician*.

[10] Nijs J., Kooning M. D., Beckwee D. (2018). Psychological distress and widespread pain contribute to the variance of the central sensitization inventory: a cross-sectional study in patients with chronic pain. *Pain Practice*.

[11] Choi Y. (2013). *An Examination of the Validity of the Central Sensitization Inventory with Chronic Disabling Occupational Musculoskeletal Disorders*.

[12] Domenech J., Sanchis-Alfonso V., López L., Espejo B. (2013). Influence of kinesiophobia and catastrophizing on pain and disability in anterior knee pain patients. *Knee Surgery, Sports Traumatology, Arthroscopy*.

[13] Tanaka K., Nishigami T., Mibu A. (2017). Validation of the Japanese version of the Central Sensitization Inventory in patients with musculoskeletal disorders. *PLoS One*.

[14] Neblett R., Hartzell M. M., Mayer T. G., Cohen H., Gatchel R. J. (2017). Establishing clinically relevant severity levels for the central sensitization inventory. *Pain Practice*.

[15] Neblett R., Cohen H., Choi Y. (2013). The Central Sensitization Inventory (CSI): establishing clinically significant values for identifying central sensitivity syndromes in an outpatient chronic pain sample. *Journal of Pain*.

[16] Cuesta-Vargas A. I., Roldan-Jimenez C., Neblett R., Gatchel R. J. (2016). Cross-cultural adaptation and validity of the Spanish central sensitization inventory. *Springer Plus*.

[17] Logan D. E., Rose J. B. (2005). Is postoperative pain a self-fulfilling prophecy? Expectancy effects on postoperative pain and patient-controlled analgesia use among adolescent surgical patients. *Journal of Pediatric Psychology*.

[18] Dimitriadis Z., Kapreli E., Strimpakos N., Oldham J. (2015). Do psychological states associate with pain and disability in chronic neck pain patients?. *Journal of Back and Musculoskeletal Rehabilitation*.

[19] Maruo T., Nakae A., Maeda L. (2013). Translation and reliability and validity of a Japanese version of the revised Short-Form McGill Pain Questionnaire (SF-MPQ-2). *Pain Research*.

[20] Hatta H., Higashi A., Yashiro H. (1998). A validation of the hospital anxiety and depression scale. *Japanese Journal of Psychosomatic Medicine*.

[21] Bot A. G. J., Becker S. J. E., Bruijnzeel H., Mulders M. A. M., Ring D., Vranceanu A.-M. (2014). Creation of the abbreviated measures of the Pain Catastrophizing Scale and the Short Health Anxiety Inventory: the PCS-4 and SHAI-5. *Journal of Musculoskeletal Pain*.

[22] Tkachuk G. A., Harris C. A. (2012). Psychometric properties of the Tampa Scale for Kinesiophobia-11 (TSK-11). *Journal of Pain*.

[23] Baron R. M., Kenny D. A. (1986). The moderator-mediator variable distinction in social psychological research: conceptual, strategic, and statistical considerations. *Journal of Personality and Social Psychology*.

[24] Mallinckrodt B., Abraham W. T., Wei M., Russell D. W. (2006). Advances in testing the statistical significance of mediation effects. *Journal of Counseling Psychology*.

[25] Preacher K. J., Hayes A. F. (2008). Asymptotic and resampling strategies for assessing and comparing indirect effects in multiple mediator models. *Behavior Research Methods*.

[26] Shimizu H. (2016). An introduction to the statistical free software HAD: suggestions to improve teaching, learning and practice data analysis. *Journal of Media, Information and Communication*.

[27] Nijs J., Torres-Cueco R., van Wilgen P. (2014). Applying modern pain neuroscience in clinical practice: criteria for the classification of central sensitization pain. *Pain Physician*.

[28] Bushnell M. C., Čeko M., Low L. A. (2013). Cognitive and emotional control of pain and its disruption in chronic pain. *Nature Reviews Neuroscience*.

[29] de Souza J. B., Potvin S., Goffaux P., Charest J., Marchand S. (2009). The deficit of pain inhibition in fibromyalgia is more pronounced in patients with comorbid depressive symptoms. *Clinical Journal of Pain*.

[30] Matsuoka H., Sakano Y. (2007). Assessment of cognitive aspect of pain; development, reliability, and validation of Japanese version of pain catastrophizing scale. *Japanese Journal of Psychosomatic Medicine*.

